# Bilateral Brachial Plexus Home Going Catheters After Digital Amputation for Patient With Upper Extremity Digital Gangrene

**DOI:** 10.4021/jocmr645w

**Published:** 2011-11-10

**Authors:** Alaa A Abd-Elsayed, John Seif, Maged Guirguis, Sherif Zaky, Loran Mounir-Soliman

**Affiliations:** a Department of Anesthesiology, University of Cincinnati, Cincinnati, Ohio, USA; b Department of Outcomes Research, Anesthesiology, Cleveland Clinic, Cleveland, Ohio, USA; c Department of Anesthesiology, Cleveland Clinic, Cleveland, Ohio, USA

## Abstract

Peripheral nerve catheter placement is used to control surgical pain. Performing bilateral brachial plexus block with catheters is not frequently performed; and in our case sending patient home with bilateral brachial plexus catheters has not been reported up to our knowledge. Our patient is a 57 years old male patient presented with bilateral upper extremity digital gangrene on digits 2 through 4 on both sides with no thumb involvement. The plan was to do the surgery under sequential axillary blocks. On the day of surgery a right axillary brachial plexus block was performed under ultrasound guidance using 20 ml of 0.75% ropivacaine. Patient was taken to the OR and the right fingers amputation was carried out under mild sedation without problems. Left axillary brachial plexus block was then done as the surgeon was closing the right side, two hours after the first block was performed. The left axillary block was done also under ultrasound using 20 ml of 2% mepivacaine. The brachial plexus blocks were performed in a sequential manner. Surgery was unremarkable, and patient was transferred to post anesthetic care unit in stable condition. Over that first postoperative night, the patient complained of severe pain at the surgical sites with minimal pain relief with parentral opioids. We placed bilateral brachial plexus catheters (right axillary and left infra-clavicular brachial plexus catheters). Ropivacaine 0.2% infusion was started at 7 ml per hour basal rate only with no boluses on each side. The patient was discharged home with the catheters in place after receiving the appropriate education. On discharge both catheters were connected to a single ON-Q (I-flow Corporation, Lake Forest, CA) ball pump with a 750 ml reservoir using a Y connection and were set to deliver a fixed rate of 7 ml for each catheter. The brachial plexus catheters were removed by the patient on day 5 after surgery without any difficulty. Patient's postoperative course was otherwise unremarkable. We concluded that home going catheters are very effective in pain control postoperatively and they shorten the period of hospital stay.

**Keywords:**

Brachial plexus; Home going catheters; Post-operative pain

## Introduction

Peripheral nerve catheter placement for pain control after painful surgical procedures has been well described. Ambulatory peripheral nerve catheters have been adopted by several institutions to facilitate recovery after discharge. Performing bilateral brachial plexus block with catheters is not frequently performed; and in our case sending patient home with bilateral brachial plexus catheters has not been reported up to our knowledge [1].

There are more reports on bilateral nerve blocks performed as an adjuvant for postoperative pain control along with general anesthesia for the surgery itself [2-4]. There are several factors that should be taken into consideration when thinking in using bilateral brachial plexus blocks. Performing bilateral upper extremity blocks caries the following disadvantages; it is time consuming, more uncomfortable to the patient, and it also carries the potential risk of increased failure rate because it usually implies using less local anesthetic solution on each side and most importantly they carry an increased risk of technique-related complications and local anesthetic toxicity. But on the other hand their use is very helpful to some cases as we will describe in our report.

## Case Report

We present a case of a patient who had severe postoperative pain after bilateral finger amputation surgery. The bilateral block technique with home going catheters was the best management for that case. Our patient was a 57 years old male patient presented with bilateral upper extremity digital gangrene on digits 2 through 4 on both sides with no thumb involvement. The patient had history of aortic stenosis and underwent aortic valve replacement 11 years ago. After 11 years, the patient came back for redo aortic valve replacement with homograft, mitral valve replacement using Cosgrove-Edward valve, and tricuspid valve repair. Postoperative course was complicated with bleeding requiring re-operation twice. The patient also developed low cardiac output state requiring high dose vasopressors that led to extremities’ ischemia, and acute renal failure (resolved later). As a result of this ischemia the patient had amputation of some of his toes bilaterally 3 months after his cardiac surgery. His fingers’ ischemia failed to improve and progressed to dry gangrene of fingers 2 to 4 on both sides requiring amputation.

The patient also had a past medical history of ankylosing spondylitis, iliac thrombophlebitis and non-autoimmune hemolytic anemia of uncertain cause.

Recent transthoracic echocardiography showed normal ejection fraction with moderate to severe mitral stenosis, with mild mitral and tricuspid regurgitation.

Given the patient significant past medical history and preoperative pain, the plan was to do the surgery under sequential axillary blocks. On the day of surgery the patient was taken to the induction room where right axillary brachial plexus block was performed under ultrasound guidance using 20 ml of 0.75% ropivacaine. Patient was taken to the OR and the right fingers amputation was carried out under mild sedation without problems. Left axillary brachial plexus block was then done as the surgeon was closing the right side, and two hours after the first block was performed. The left axillary block was done also under ultrasound guidance using 20 ml of 2% mepivacaine. The brachial plexus blocks were performed in a sequential manner to avoid simultaneous local anesthetic peaks which could reach toxic level and helped to decrease the risk of local anesthetic related side effects

Surgery was unremarkable, and patient was transferred to the post anesthetic care unit in stable condition. Over that first postoperative night, the patient complained of severe pain at the surgical sites with minimal pain relief with parenteral opioids. Patient was brought back to the block room next morning where we placed bilateral brachial plexus catheters (right axillary and left infra-clavicular brachial plexus catheters). We decided not to place bilateral infra-clavicular due to anatomical difficulty on right side secondary to the presence of Hickman catheter in the right infraclavicular area. Bilateral interscalene or supraclavicular blocks were excluded as an option to avoid the possibility of bilateral phrenic nerve palsies [5,6]. The right axillary block was performed under ultrasound guidance and a total of 15 ml of 0.75% ropivacaine was injected, then a catheter was placed and tunneled to the right shoulder area. Left infraclavicular block was performed also under ultrasound guidance and a total of 15 ml of 0.75 % ropivacaine was used. The catheter was placed and tunneled in the left pectoral area. The patient reported significant pain relief after the blocks were performed. Ropivacaine 0.2% infusion was started at 7 ml per hour basal rate only with no boluses on each side. The patient's pain continued to be well controlled over the second postoperative day with the peripheral nerve catheter infusions, so the patient was discharged home with the catheters in place after receiving the appropriate education. On discharge both catheters were connected to a single ON-Q (I-flow Corporation, Lake Forest, CA) ball pump with a 750 ml reservoir using a Y connection and were set to deliver a fixed rate of 7 ml for each catheter. We used fixed rate pump as the patient after finger amputation will not be able to use bolus button. Over the next few days the patient continued to do well in term of his pain and was followed up by the acute pain management service through a daily phone call. The pump was switched by a home-visiting nurse on post-discharge day 3. The brachial plexus catheters were removed by the patient on day 5 after surgery without any difficulty. Patient's postoperative course was otherwise unremarkable.

## Discussion

There are few reports on bilateral plexus blocks in the literature. A bilateral technique takes time to accomplish, and it theoretically doubles the amount of any discomfort the patient may experience during a single technique. Potentially, it carries a lesser success rate and an increased risk of side effects and local anesthetic toxicity. The interscalene block carries a high risk of injuring the phrenic nerve [5,6]. Bilateral supra-clavicular blocks are avoided because of the risk of overwhelming respiratory compromise due to bilateral diaphragmatic paralysis and the potential for pneumothorax. Bilateral blocks of any kind must be assessed carefully on a case-by-case basis [7].

Our case is unique in that we performed infraclavicular brachial plexus block on one side with axillary block on the other side to avoid the risk (although small under ultrasound guidance) of bilateral pneumothoracies, specially in the light of the anatomical alterations and vascular deformities in our case

The infraclavicular brachial plexus block with catheter is potentially less amenable to be dislodged and might have less risk of infection. While axillary block is easier to perform, placing a catheter in this location might be unstable due to arm movements, and the risk of infection is probably higher.

The choice of home going pumps for ambulatory peripheral nerve catheters should be mainly based on the intended regimen of infusion used by each individual institution. There are few studies comparing various infusion strategies (basal only versus patient controlled boluses). Currently there are no guidelines for the optimal drug or rate of infusions for these catheters. The coast of these pumps, simplicity as well as their accuracy and reliability are also important factors in making this choice [8]. In our case, the patient inability to deliver a bolus dose eliminated one of these factors. We elected to use the ON-Q ball pump that is very simple to use with the least need for patient's interference. Also, the large volume reservoir bag and dual infusion tubing allowed drug delivery to both sides for few days without the need of two pumps.

One major concern when sending patients home with continuous infusion of local anesthetics is the total volume and dose infused per hour with no reported cases of systemic toxicity for this novel mode of analgesia. The maximum dose to be infused at home of ropivacaine through a peripheral nerve catheter is still to be determined. Patients can recover safely and stay pain-free at home after surgery, avoiding the risk of addiction to narcotics and of hospital-acquired infections, while saving medical costs by reducing the length of hospital stays. This case represents the success and convenience in using the home going catheters for pain control.

### Conclusion

Home going catheters are very effective in pain control postoperatively and they shorten the period of hospital stay.
